# COVID-19 and suicide in Japan from March 2020 to February 2023

**DOI:** 10.1038/s41598-026-52517-4

**Published:** 2026-05-20

**Authors:** Quentin Batista, Daisuke Fujii, Taisuke Nakata, Takeki Sunakawa

**Affiliations:** 1 Amazon Japan Inc., 1-8-1 Shimomeguro, Tokyo, Tokyo 153-0064 Japan; 2 International Monetary Fund, 700 19th St. NW, Washington, DC 20431 USA; 3 Faculty of Economics, University of Tokyo, 7-3-1 Hongo, Tokyo, Tokyo 113-0033 Japan; 4 Department of Economics, Hitotsubashi University, 2-1 Naka, Kunitachi, Tokyo 186-8601 Japan

**Keywords:** Diseases, Health care, Medical research, Psychology, Psychology, Risk factors

## Abstract

We estimate excess mortality due to suicides during the COVID-19 pandemic in Japan and the extent to which the increase in unemployment can explain it. Using a time-series model, as well as pre-COVID private-sector forecasts of the unemployment rate, we find that the COVID-19 crisis increased suicides in Japan by approximately 11,000 from March 2020 to February 2023. Furthermore, the increase in unemployment can account for less than 10 percent of this increase. We also find that the excess deaths due to suicides are skewed towards younger generations and females and that lost years of life expectancy associated with the excess deaths due to suicide are almost as large as those associated with COVID-19 deaths.

## Introduction

**Figure 1:**
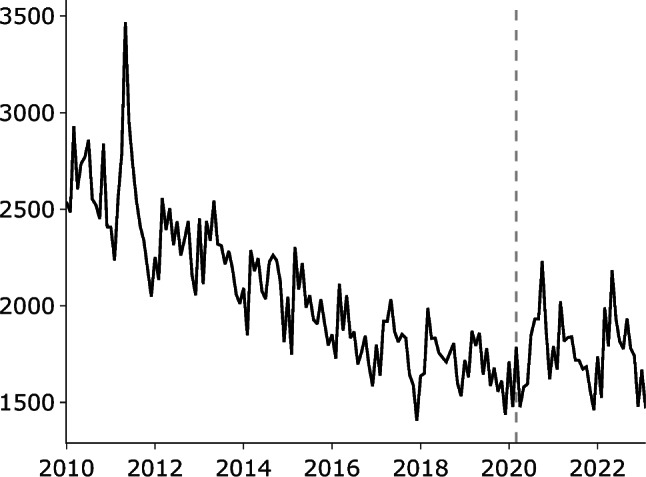
The number of suicides in Japan. Source: Ministry of Health, Labor, and Welfare.

A large number of people died from infection during the COVID-19 pandemic. In Japan, there was another notable tragedy—a surge in suicides. As shown in Fig. 1, the number of suicides was on a steady path of decline for more than a decade before the COVID-19 crisis intensified in the spring of 2020. However, once the COVID-19 crisis began to affect every aspect of our lives, the number of suicides sharply increased. Suicides stayed above the downward trend of previous years throughout the crisis.

In this paper, we estimate the number of excess deaths due to suicide during the COVID-19 pandemic and the extent to which the increase in unemployment can explain it. Utilizing the fact that the number of suicides is strongly correlated with the unemployment rate, we use private-sector forecasts of the unemployment rate before the COVID-19 crisis began to estimate the hypothetical path of suicides that would have prevailed in the absence of the COVID-19 crisis. We then interpret the difference between the actual and hypothetical numbers of suicides as the number of excess deaths due to suicide during the COVID-19 pandemic. We also compute another hypothetical path of suicides that would be predicted from our model under the actual path of the unemployment rate. We interpret the difference between these two hypothetical paths of suicides as part of the excess deaths due to suicide explained by the increase in the unemployment rate.

According to our baseline specification, we find that the number of excess deaths due to suicide from March 2020 to February 2023 is 10,800. The increase in the unemployment rate can explain less than 10 percent of the excess mortality due to suicides; the remaining excess mortality due to suicide can be interpreted as being driven by pandemic-specific factors beyond the typical economic hardship associated with a standard recession.

We also find that female suicides account for about half of the excess mortality due to suicide. Prior to the pandemic, female suicides accounted for only about 30% of suicides. Thus, the excess-suicide ratio—the proportion of excess deaths due to suicide to the overall suicide—is higher for females than for males. The excess-suicide ratio tends to be higher for younger generations, especially for females. The years of life lost associated with excess mortality due to suicide are almost as large as those associated with COVID-19 deaths.

We consider several alternative estimates that differ in the starting date of estimation. The estimate of excess deaths due to suicide ranges from around 7000 to around 13,000. Regardless of the estimation period, the increase in the unemployment rate can explain a small fraction of the excess mortality due to suicide. Regardless of the estimation period, the excess-suicide ratio tends to be higher for females and younger generations than for males and older generations.

To design policy to reduce excess mortality due to suicide in a pandemic, it is crucial for policymakers to first understand the extent to which suicide deviated from the pre-pandemic trend during the COVID-10 pandemic and how that increase is tied to worsening economic situations during the pandemic. The results of our paper suggest that most of the excess mortality due to suicide cannot be explained by a typical relationship between unemployment rate and suicide, suggesting a need for policy prescriptions that are different from those taken in a typical recession.

Commentators list various reasons for this increase in suicide during the COVID-19 crisis. Those reasons include, but are not limited to, economic hardship, loneliness associated with reduced in-person interactions, suicides triggered by the suicides of celebrities, and domestic violence cases that are likely caused by sudden adjustments in how families live together. Anecdotal evidence suggests that the suicides of a few prominent actors and actresses nontrivially contributed to the rise of suicides in the fall of 2020. Our analysis shows that economic hardship associated with a typical economic decline cannot explain much of the excess mortality due to suicide, but is silent about the extent to which these other factors contributed to excess mortality due to suicide. Such an investigation is immensely important, but it is outside the scope of our paper.

However, it is crucial for the government to take measures to prevent increases in suicides and take note of the fact that well-intended policies to control infection could potentially contribute to excess mortality due to suicide in future pandemics.

Our paper is related to the existing work on suicides during the COVID-19 crisis in Japan. Some papers document either the initial decline in suicides in spring 2020 or the subsequent increase in suicides in the second half of 2020 in Japan.^1–6^ Other papers analyze the suicide trend in a more extended sample through September 2021, or use regional variation in the unemployment rate and the number of suicides to estimate the elasticity of suicides to fluctuations in the unemployment rate in the first six months of the COVID-19 crisis.^7,8^ Our paper differs from these papers because we estimate the extent to which the rise in unemployment can account for excess mortality due to suicide.

Our paper is also related to studies investigating the socio-economic impacts of the COVID-19 crisis in Japan. Some papers examine how the COVID-19 crisis affected labor markets.^9–14^. Beyond labor markets, researchers have examined a wide range of outcomes during the crisis, ranging from consumption, GDP, and education to marriage and birth.^15–21^ Methodologically, our paper is closely related to those that use the difference between actual and predicted outcomes based on a time-series model to estimate the impact of COVID-19 on labor markets.^22,23^

## Method

### Idea

In estimating the hypothetical number of suicides in the absence of COVID-19, we use the fact that the number of suicides and the unemployment rate are strongly correlated in Japan. Figure 3 plots suicides against the unemployment rate at a monthly frequency since 2010. This correlation is robust to different sample periods and is well-known.^24^

Thus, if we knew the path of the unemployment rate that would have prevailed in the absence of the COVID-19 crisis, we could use this relationship between suicides and the unemployment rate to estimate the hypothetical number of suicides that would have prevailed in the absence of the COVID-19 crisis. We use the pre-COVID private-sector forecasts of the unemployment rate as an approximation of such a hypothetical path of the unemployment rate.

**Figure 2:**
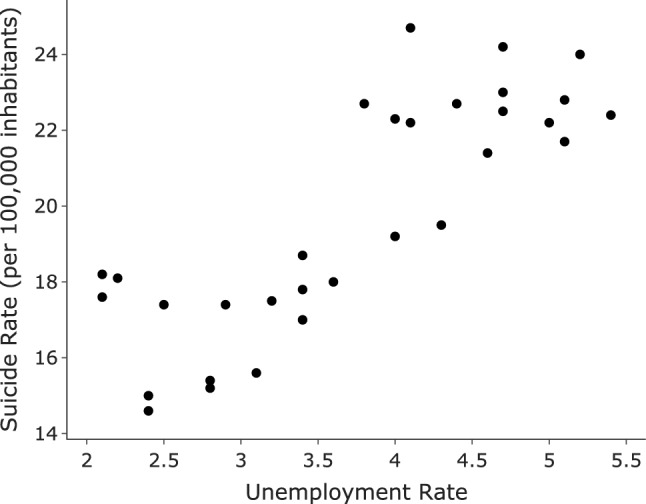
Pre-COVID unemployment rate projections. Source: Statistics Bureau, Ministry of Internal Affairs and Communications; Pre-COVID projections are based on the authors’ calculation using the private-sector forecasts of the unemployment rate. (See Table 1 for details.).

As shown in Fig. 2, and as discussed in detail in Section 3, prior to the COVID-19 crisis, private-sector analysts predicted that the unemployment would hover slightly below 2.4 percent in 2020 and 2021. The actual unemployment rate has been, on average, 0.45 percentage points above the projection.

### Model

We use a monthly time interval. We specify our model at the following disaggregate level: two genders (male and female) and eight age groups (ages 0–19, 20–29, 30–39, 40–49, 50–59, 60–69, 70–79, and 80–). For each age and gender group, we assume the following relationship between the number of suicides and the unemployment rate:

1 $$\begin{aligned} S_{a,g,t} = \sum _{m=1}^{12}\left( \alpha _{a,g,m}+\beta _{a,g,m}t\right) \delta _{m}+\gamma _{a,g}U_{a,g,t}+\varepsilon _{a,g,t} \end{aligned}$$

where $S_{a,g,t}$ is the total number of suicides for individuals in age group *a* of gender *g* at time *t*. $U_{a,g,t}$ is the unemployment rate for individuals in age group *a* of gender *g* at time *t*. $\delta _{m,t}$ is a month *m* dummy variable at time *t*. That is, $\delta _{m,t}=1$ if time *t* is at month $m\in \{\text {January},\text {February},...,\text {December}\}$ of any year, and $\delta _{m,t}=0$ otherwise. $\alpha _{a,g,m}+\beta _{a,g,m}t$ is an age, gender, and month specific intercept and time trend. $\varepsilon _{a,g,t}$ is an i.i.d. error term.

We estimate the model parameters for each group of age and gender equation-by-equation using Ordinary Least Squares. We use data in the pre-COVID period from January 2010 to February 2020 for the estimation. With the estimated parameters $\{\left( \hat{\alpha }_{a,g,m}\right) _{m=1}^{12},\left( \hat{\beta }_{a,g,m}\right) _{m=1}^{12},\hat{\gamma }_{a,g}\}$ from the pre-COVID period at hand, we compute the hypothetical number of suicides during the COVID-19 crisis using pre-COVID unemployment forecasts. Specifically, we compute *the number of suicides consistent with pre-COVID unemployment forecasts*,

2 $$\begin{aligned} \hat{S}_{a,g,t}^{F}=\sum _{m=1}^{12}\left( \hat{\alpha }_{a,g,m}+\hat{\beta }_{a,g,m}t\right) \delta _{m}+\hat{\gamma }_{a,g}U_{a,g,t}^{F} \end{aligned}$$

by using the projected unemployment rate $U_{a,g,t}^{F}$ for each group of age *a* and gender *g* at time *t*. We attribute the difference between the actual number of suicides and the hypothetical number of suicides, $S_{a,g,t}-\hat{S}_{a,g,t}^{F}$, to the COVID-19 crisis. We also compute *the number of suicides consistent with the actual unemployment rate*,

3 $$\begin{aligned} \hat{S}_{a,g,t}=\sum _{m=1}^{12}\left( \hat{\alpha }_{a,g,m}+\hat{\beta }_{a,g,m}t\right) \delta _{m}+\hat{\gamma }_{a,g}U_{a,g,t} \end{aligned}$$

by using the actual unemployment rate $U_{a,g,t}$ at time *t*. Then $\hat{S}_{a,g,t}-\hat{S}_{a,g,t}^{F}$ is the number of suicides that can be explained by the increase in the unemployment rate. $S_{a,g,t}-\hat{S}_{a,g,t}=\hat{\varepsilon }_{a,g,t}$ is an estimate of the residual of Equation (1) and the number of suicides that cannot be explained by the economic hardship associated with a typical recession.

## Data

In this section, we briefly describe the suicide and unemployment data we use. See Table 1 for the source of the original data (Panel A) and the list of institutions publishing unemployment forecasts (Panel B).

**Table 1:** Data source.

Data	Frequency	Description	source
Suicides	Monthly	Number of suicides for different gender and age groups	MHLW
Unemployment	Monthly	Unemployment rates for different gender and age groups	Statistics Bureau
COVID-19	Weekly	Number of deaths due to COVID-19	National Institute of Population
infection deaths		For different gender and age groups	and Social Security Research
Life expectancy	–	Life expectancy for different gender and age groups	MHLW

Name of institution	Release date	Frequency	
Nomura Holdings	November 11, 2019	Quarterly	
Sumitomo Mitsui Trust Bank	November 22, 2019	Semi-annual	
Nisseikiso Research Institute	December 8, 2019	Quarterly	
SMBC Nikko Securities	December 9, 2019	Quarterly	
Daiichi Seimeikeizai Research Institute	December 9, 2019	Annual	
Japan Center for Economic Research	December 9, 2019	Quarterly	
Mitsubishi UFJ Research & Consulting Co., Ltd.	December 9, 2019	Quarterly	
Daiwa Institute of Research Ltd.	December 9, 2019	Quarterly	
Mizuho Information & Research Institute	December 9, 2019	Quarterly	
Shinkin Central Bank Research Institute	December 11, 2019	Annual	
Shinsei Bank	December 24, 2019	Semi-annual	
Teikoku Databank	January 8, 2020	Annual	
Japan Research Institute, Limited	February 4, 2020	Quarterly	

We collect the latest forecast by each institution before February 15, 2020. Some institutes release only semi-annual or annual forecasts. We interpolate these forecasts to make quarterly forecasts. Also, some institutes release no forecasts for 2022 and 2023. We use the same value of the forecast for the fourth quarter of 2021 to make 2023 forecasts.

**Figure 3:**
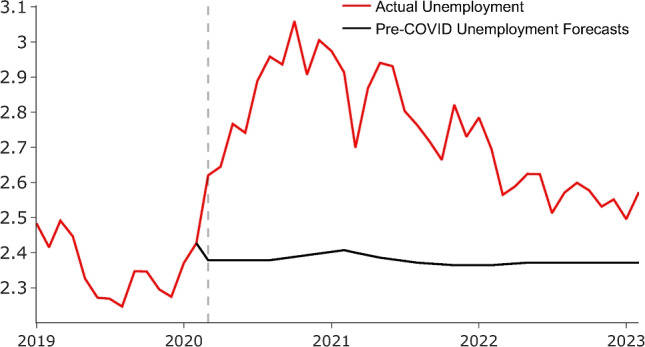
Suicides and the Unemployment Rate. Source: Ministry of Health, Labor, and Welfare; Statistics Bureau, Ministry of Internal Affairs and Communications.

### Suicides

We collect monthly suicide data from the Ministry of Health, Labor, and Welfare (MHLW) for different genders and age groups. The suicide data are obtained from https://www.mhlw.go.jp/stf/seisakunitsuite/bunya/0000140901.html. We make the following two adjustments to the original data. First, only provisional values of suicide data are available at a monthly frequency, whereas confirmed values are available at an annual frequency. Given that we observe a significant difference between provisional and confirmed values, we make an adjustment to the provisional values to bridge this gap. Second, the age of some suicide victims is not available. Therefore, when we have some data points for gender *g* in month *m* with unknown age, we divide these data points into each age group by using the proportion of provisional suicides for gender *g* and known age group *a* in month *m*.

### Actual unemployment

We collect monthly unemployment data from the Statistics Bureau of Japan for different genders and age groups. The age groups that are available for unemployment do not match those available for suicide data. We use a simple weighting scheme described in the Appendix to construct unemployment rate series for the suicide age groups. We assume that the unemployment rate is zero for individuals younger than 15 and older than 70.

**Figure 4:**
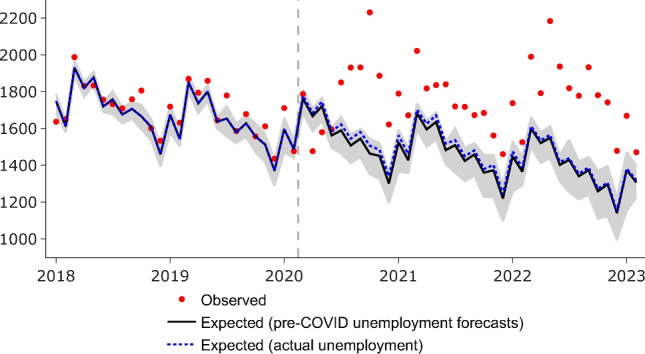
Actual and hypothetical suicides. Source: Ministry of Health, Labor, and Welfare; the expected number of suicides (-COVID and Pre-COVID) are based on the authors’ calculation..

### Pre-COVID unemployment forecasts

We collect forecasts for the aggregate unemployment rate published by various institutions. Panel B of Table 1 shows the frequency, the institution name, and the release date of forecasts. We utilize the latest forecasts from each institution in the pre-COVID period, issued at a quarterly frequency. Some institutes release only semi-annual or annual forecasts. We interpolate these forecasts to make quarterly forecasts. Also, some institutes release no forecasts for 2022. We use the same value of the forecast for the fourth quarter of 2021 to make 2022 forecasts. We then compute the mean of all the quarterly forecasts. Finally, we use some interpolation techniques to construct our monthly measures, which are denoted by $U_{t}^{F}$ and shown by the black line in Fig. 3 above. We attribute the quarterly forecast to the middle month for each quarter, and use continuous piece-wise linear interpolation.

**Table 2:** Sensitivity analysis.

		Contribution from		Excess-suicide ratio		Years of
Estimation period	Excess deaths due to suicide	Unemployment	Total	Male	Female	Life lost
2010–2019	12,889	12.0%	0.20	0.17	0.28	494,775
2011–2019	12,353	8.6%	0.19	0.14	0.29	484,016
2012–2019*	10,849	7.7%	0.17	0.13	0.26	406,952
2013–2019	10,919	7.4%	0.17	0.12	0.28	407,967
2014–2019	11,001	0.0%	0.17	0.13	0.27	416,222
2015–2019	8679	2.9%	0.14	0.09	0.22	313,009
2016–2019	6264	0.8%	0.10	0.04	0.22	257,447
2017–2019	7091	1.3%	0.10	0.05	0.23	284,724

While we wish we had age-gender specific unemployment forecasts $U_{a,g,t}^{F}$ for the COVID-19 period to estimate the hypothetical number of suicides $\hat{S}_{a,g,t}^{F}$ using Equation (2), forecasts for the aggregate unemployment rate are only available in data. We construct age-gender-specific unemployment forecasts as out-of-sample predictions of an estimated regression of age-gender-specific unemployment on aggregate unemployment. Specifically, we estimate

4 $$\begin{aligned} U_{a,g,t}=c_{a,g,0}+c_{a,g,1} t+c_{a,g,2}U_{t}+e_{a,g,t} \end{aligned}$$

to obtain age-gender specific parameters $\{\hat{c}_{a,g,0},\hat{c}_{a,g,1},\hat{c}_{a,g,2}\}$ by using the age-gender specific unemployment rate $U_{a,g,t}$ and the aggregate unemployment rate $U_{t}$ in the pre-COVID period from January 2012 to February 2020. $e_{a,g,t}$ is an i.i.d. error term. Then we compute

5 $$\begin{aligned} U_{a,g,t}^{F}=\hat{c}_{a,g,0}+\hat{c}_{a,g,1}t+\hat{c}_{a,g,2}U_{t}^{F} \end{aligned}$$

by using the mean forecast for the aggregate unemployment rate $U_{t}^{F}$ for the COVID-19 period.

## Results

Figure 4 shows the actual and hypothetical numbers of suicides. The red dots show the actual number of suicides, while the solid black line shows the hypothetical number of suicides consistent with pre-COVID unemployment forecasts. The shadow area around the solid black line is the one-standard-deviation confidence interval of the estimate.

The actual number of suicides exceeds the hypothetical number of suicides, except for April and May 2020. The number of actual suicides is outside of the confidence interval during most of the COVID-19 crisis, whereas it is inside the confidence interval in the pre-COVID period. In particular, the number of excess deaths due to suicide was large in the second half of 2020. The total number of excess deaths due to suicide is 10,848 for the three years from March 2020 to February 2023.

The dashed blue line represents the number of suicides consistent with what the model would predict based on the actual path of the unemployment rate. The dashed blue line is somewhat above the solid black line, indicating that the increase in the unemployment rate during the COVID-19 crisis can account for some of the excess mortality due to suicides. However, a large portion of the excess mortality due to suicide is left unexplained. We find that 840—about 8 percent—of 10,848 excess deaths due to suicide can be explained by the increase in the unemployment rate; the remaining excess deaths due to suicide cannot be explained by the economic hardship associated with a typical recession. Even in the analysis that excludes those aged below 20 and above 69, we find that only 10 percent of excess deaths due to suicide during the COVID-19 pandemic can be explained by the increase in unemployment.

Our decomposition is based on the assumption that the past relationship between the unemployment rate and suicides did not significantly change during the COVID-19 crisis. Thus, our estimate of ”excess deaths due to suicide that the rise in the unemployment rate cannot explain” includes excess deaths due to suicide that might have been related to the rise in the unemployment rate during the COVID-19 crisis, but would not have occurred in other episodes with a similar rise in the unemployment rate. In other words, we classify the effect of a possible change in the elasticity of suicides to the unemployment fluctuation as the effect that cannot be explained by ”normal” unemployment fluctuations.

**Figure 5:**
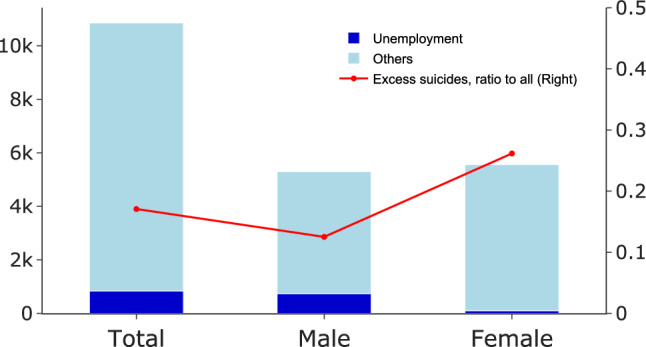
Distribution of excess deaths due to suicide: gender. Source: Authors’ calculation.

Figure 5 shows the distribution of excess deaths due to suicide by gender. Male and female excess deaths due to suicide are about the same, with female excess deaths due to suicide being slightly larger than male suicides. This result is striking because the number of suicides had been consistently lower for females than for males in Japan prior to the COVID-19 crisis. The red line shows the ratio of excess deaths due to suicide to overall suicides—including those not associated with the COVID-19 crisis, according to our model. The ratio is 0.13 for males, whereas it is 0.26 for females. This comparison reinforces the sense in which the COVID-19 crisis impacted female suicides more than male suicides. Finally, according to the dark blue bars, almost none of the excess female suicides can be explained by the increase in the unemployment rate for females.

The previous research on excess mortality due to suicide during the COVID-19 pandemic in Japan—those referenced in the introduction—has also documented a significant increase in suicide among young women. Our marginal contributions to these bodies of work are two-fold. The first is to document the role of the unemployment rate in explaining the increase in suicide among young women. The second is to confirm the robustness of the significant increase in suicide among young women over a longer time horizon than the previous research.

**Figure 6:**
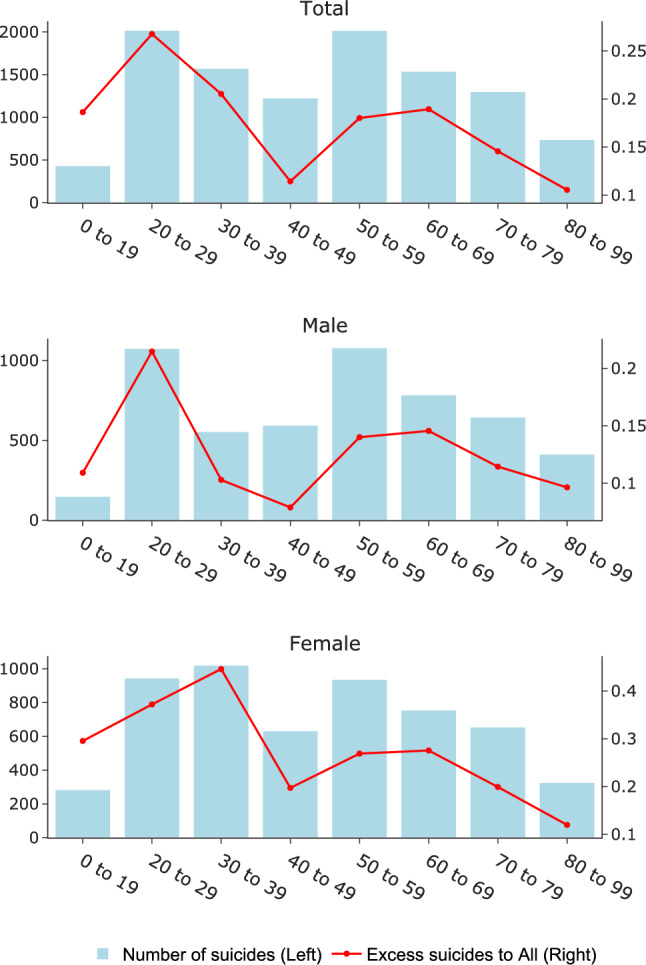
Distribution of excess deaths due to suicide: age.

Light blue bars in Fig. 6 show the distribution of excess deaths due to suicide across different age groups: The top panel is for total (males and females combined), whereas the middle and bottom panels are for males and females, respectively. Excess deaths due to suicide for 20s and 50s are larger than other age groups for total and males. For females, excess deaths due to suicide for 30s are the largest for 30s, followed by 20s and 50s.

The red line shows the excess-suicide ratio. Overall, this excess-suicide ratio tends to be higher for younger generations. The excess-suicide ratio for the total is the highest for the 20s, followed by the 30s. The ratio for males is the highest for the 20s, followed by the 60s. The ratio for females is the highest for the 30s, followed by the 20s. All in all, the COVID-19 crisis impacted suicides for younger generations more than for older generations.

**Figure 7:**
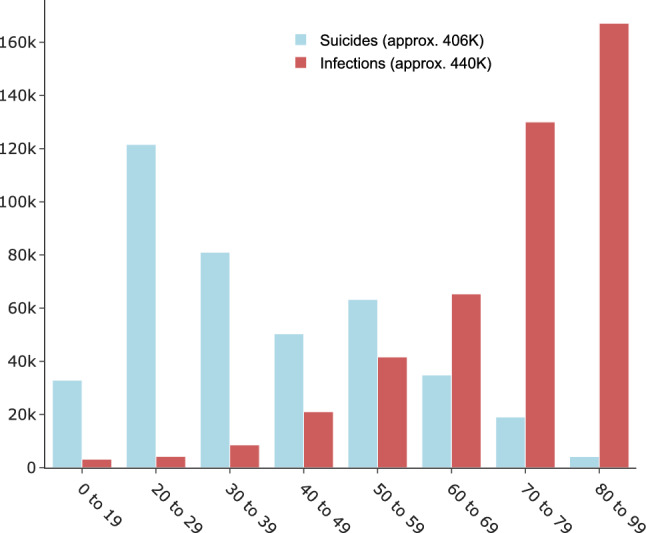
Distribution of excess deaths due to suicide and COVID-19 deaths: years of life lost.

Finally, Fig. 7 shows the distribution of excess deaths due to suicide and COVID-19 deaths in terms of the expected years of life lost across ages. We calculate the years of life expectancy for each age-gender group, multiply them by the number of excess deaths due to suicide or the number of COVID-19 deaths for each age-gender group, and aggregate them across genders.

Because younger generations have longer life expectancies than older generations and the distribution of excess deaths due to suicide is relatively flat for most age groups, the distribution of years of life lost associated with excess deaths due to suicide is skewed towards younger generations, as shown by light blue bars. On the other hand, the distribution of COVID-19 deaths skewed towards older generations, as shown by red bars. Because the fatality rate increases with age for COVID-19, the number of COVID-19 deaths was larger for older generations than for younger generations. Although their life expectancies are shorter than those of younger generations, the larger number of deaths dominates, resulting in a skewed distribution of years of life lost towards older generations. When aggregated across age groups, the expected years of life lost associated with excess mortality due to suicide (approximately 406 thousand years) are similar to those from COVID-19 deaths (approximately 440 thousand years).

## Sensitivity analysis

In this section, we discuss how our estimate depends on the choice of the estimation period. In our baseline specification, we estimated our model using data from 2012 to 2019. Here, we consider seven alternative starting years for the estimation period. Table 2 illustrates how our key estimated statistics vary with the choice of the estimation period.

According to Table 2, the estimate of excess suicides is larger when the starting year of the estimation period is earlier. This result arises because the pace of the secular decline in suicide slows down over time. An earlier starting year of the estimation period means a more negative estimate for the coefficient on the time trend variable, resulting in a lower predicted path of the number of suicides in the absence of the COVID-19 crisis—and a higher estimate of excess deaths due to suicide. The estimate ranges from 7091 to 12,889.

**Figure 8:**
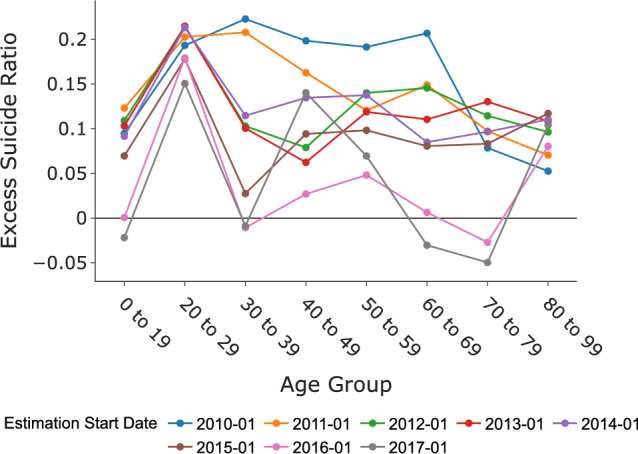
Sensitivity analysis: the excess-suicide ratio across age groups for male.

Regardless of the estimation period, the contribution from the rise in unemployment is small, ranging from 0.0% (2014–2019 estimation period) to 12.0% (2010–2019 estimation period). For all estimation samples, the excess-suicide ratio is higher for females than for males. Similarly to the excess mortality due to suicide, lost life years are larger when the starting year of the estimation period is earlier. The estimated loss of life years ranges from 284,724 to 494,775.

**Figure 9:**
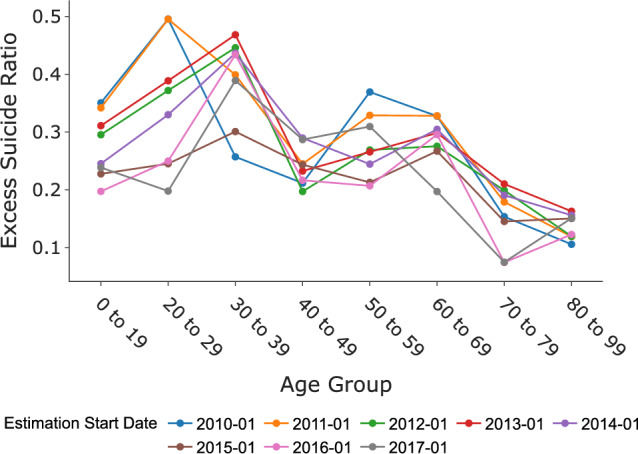
Sensitivity analysis: the excess-suicide ratio across age groups for female.

Finally, Figs. 8 and 9 illustrate how the excess-suicide ratios for different age groups vary according to the estimation period. Figure 9 is for males, whereas Fig. 9 is for females. Regardless of the estimation period, the excess-suicide ratio tends to be higher for younger generations than for older generations for both males and females, and this tendency is more salient for females than for males.

## Limitations

In decomposing the excess mortality due to suicide into parts that can and cannot be explained by the increase in the unemployment rate, we are implicitly assuming that the relationship between the unemployment rate and suicide is stable over time. However, it is plausible that the relationship between them during the COVID-19 pandemic was different from that during a normal time. Thus, our decomposition exercise is decomposing the excess mortality due to suicide into parts that can and cannot be explained by what the increase in the unemployment rate would imply if the relationship between the unemployment rate and suicide were stable. Investigating the relationship between the unemployment rate and suicide during the COVID-19 pandemic would likely require more sophisticated analysis involving micro-level data, which we leave for future research.

The relationship between the unemployment rate and suicide in our analysis is based on an aggregate time-series relationship, and is correlational—not causal. In the literature, there are several rigorous empirical analyses of the relationship between the unemployment rate and suicide in Japan based on differences-in-differences and/or event-study analyses^8,25^. However, being an observational panel analysis, the evidence for causality is not definitive. Outside Japan, there are some empirical analyses examining the causality from unemployment rate to suicide, taking advantage of exogenous variations in the unemployment rate caused by plant closures and mass layoffs^26–28^. These studies have found some evidence for the causality from unemployment to suicide.

Though our statistical model is relatively simple, it does a reasonably good job in an out-of-sample forecasting exercise in which we set the training period to 2010:01-2017:02 and the test period to 2017:03-2020:02. Nevertheless, caution is needed in taking the estimated excess mortality due to suicide from any one specification of the model at face value. For that reason, we conducted a robustness analysis based on different sub-samples in the ”Sensitivity Analysis” section. The range of values in that robustness analysis tells us the degree of uncertainty surrounding our baseline estimate.

## Conclusion

We estimated the number of excess deaths due to suicide during the COVID-19 pandemic and the extent to which the increase in unemployment can explain it. Using a time-series model, as well as pre-COVID private-sector forecasts of the unemployment rate, we found that the COVID-19 crisis increased suicides in Japan by approximately 11,000 from March 2020 to February 2023. Furthermore, the increase in unemployment accounted for less than 10 percent of this increase. We also found that the excess mortality due to suicide are skewed towards younger generations and females and that lost years of life expectancy associated with the excess mortality due to suicide are almost as large as those associated with COVID-19 deaths.

In this paper, we focused on estimating the number of excess deaths due to suicides during the COVID-19 pandemic. A natural next step would be to investigate why the COVID-19 crisis led to a surge in suicides and what could have been done to mitigate the surge. Such an investigation is likely to be beneficial for policymakers preparing for the next pandemic.

## Data Availability

The data used in this study are all publicly available. Replication codes are available upon request. Requests should be directed to Taisuke Nakata (taisuke.nakata@e.u-tokyo.ac.jp).

## References

[1] Anzai, T., Fukui, K., Ito, T., Ito, Y. & Takahashi, K. Excess mortality from suicide during the early COVID-19 pandemic period in Japan: A time-series modeling before the pandemic. *J. Epidemiol.* **31**(2), 152–156 (2021). 33310986 10.2188/jea.JE20200443 PMC7813773

[2] Tanaka, T. & Okamoto, S. Increase in suicide following an initial decline during the COVID-19 pandemic in Japan. *Nat. Hum. Behav.* **5**, 229–238 (2021). 33452498 10.1038/s41562-020-01042-z

[3] Eguchi, A. et al. Suicide by gender and 10-year age groups during the COVID-19 pandemic vs previous five years in Japan: An analysis of national vital statistics. *Psychiatry Res.* **305**, 114–173 (2021). 10.1016/j.psychres.2021.114173 PMC8763632 34469804

[4] Nomura, S. et al. Trends in suicide in Japan by gender during the COVID-19 pandemic, through December 2020. *Psychiatry Res.* **300**, 229–238 (2021). 10.1016/j.psychres.2021.113913 PMC9068581 33839422

[5] Sakamoto, H., Ishikane, M., Ghaznavi, C. & Ueda, P. Assessment of suicide in japan during the covid-19 pandemic vs previous years. *JAMA Network Open*. **2** (2021). 10.1001/jamanetworkopen.2020.37378 PMC7856546 33528554

[6] Ueda, M., Nordstrom, R. & Matsubayashi, T. Suicide and mental health during the COVID-19 pandemic in Japan. *J. Public Health*. **2** (2021). 10.1093/pubmed/fdab113 PMC8083330 33855451

[7] Horita, N. & Moriguchi, S. Trends in suicide in Japan following the 2019 coronavirus pandemic. *JAMA Netw. Open* **5**, e224739–e224739 (2022). 35348713 10.1001/jamanetworkopen.2022.4739 PMC8965634

[8] Ando, M. & Furuichi, M. The association of COVID-19 employment shocks with suicide and safety net use: An early-stage investigation. *PLoS One* **17**, e0264829 (2022). 35324902 10.1371/journal.pone.0264829 PMC8947077

[9] Kikuchi, S., Kitao, S. & Mikoshiba, M. Who suffers from the COVID-19 shocks? labor market heterogeneity and welfare consequences in Japan. *J. Japan. Int. Econ.* **59**, 101117 (2021).

[10] Hoshi, K., Kasahara, H., Makioka, R., Suzuki, M. & Tanaka, S. Trade-off between job losses and the spread of COVID-19 in Japan. *Japan. Econ. Rev.* **72**, 683–716 (2021). 10.1007/s42973-021-00092-w PMC8384925 34456605

[11] Fukui, M., Kikuchi, S. & Goalist Co, L. Job creation during the COVID-19 pandemic in Japan. *CREPE Discussion Paper*. **73** (2020).

[12] Morikawa, M. Productivity of working from home during the COVID-19 pandemic: Panel data analysis. *RIETI Discussion Paper Series* (2021).

[13] Kawaguchi, D., Kitao, S. & Nose, M. The impact of COVID-19 on Japanese firms: Mobility and resilience via remote work. *RIETI Discussion Paper Series* (2021). 10.1007/s10797-022-09749-7 PMC9440746 36092538

[14] Kawaguchi, D. & Motegi, H. Who can work from home? The roles of job tasks and hrm practices. *J. Japan. Int. Econ.* **62**, 101162 (2021).

[15] Kikuchi, J., Nagao, R. & Nakazono, Y. Fear of COVID-19 contagion: Heterogeneous responses of consumption to COVID-19. *ISER DP* (2021).

[16] Watanabe, T. & Omori, Y. Online consumption during and after the COVID-19 pandemic: Evidence from Japan. *CARF Working Paper Series* (2021).

[17] Takaku, R. & Yokoyama, I. What the COVID-19 school closure left in its wake: Evidence from a regression discontinuity analysis in Japan. *J. Public Econ.* **195**, 104364 (2021). 33437102 10.1016/j.jpubeco.2020.104364 PMC7791313

[18] Ikeda, M. & Yamaguchi, S. Online learning during school closure due to COVID-19. *Japan. Econ. Rev.* **72**, 471–507 (2021). 10.1007/s42973-021-00079-7 PMC8190173 34127905

[19] Yamamura, E. & Tsustsui, Y. School closures and mental health during the COVID-19 pandemic in Japan. *J. Populat. Econ.* **34**, 1261–1298 (2021). 10.1007/s00148-021-00844-3 PMC8184870 34121815

[20] Asakawa, S. & Ohtake, F. Impact of temporary school closure due to covid-19 on the academic achievement of elementary school students. *Graduate School of Economics and Osaka School of International Public Policy (OSIPP) Osaka University Discussion Papers In Economics And Business* **21**, 1–84 (2021).

[21] Naito, T. & Ogawa, H. COVID-19, self-restraint at home, and pregnancy: Evidence from Japan. *Appl. Econ. Lett*. 1–4 (2021).

[22] Fukai, T., Ichimura, H. & Kawata, K. Describing the impacts of COVID-19 on the labor market in Japan until June 2020. *Japan. Econ. Rev.* **72**, 439–470 (2021). 10.1007/s42973-021-00081-z PMC8286989 34305434

[23] Fukai, T., Ikeda, M., Kawaguchi, D. & Yamaguchi, S. COVID-19 and the employment gender gap in Japan. *J. Japan. Int. Econ.* **68**, 101256 (2023). 37021061 10.1016/j.jjie.2023.101256 PMC9995392

[24] Chen, J., Choi, Y. J. & Sawada, Y. How is suicide different in Japan?. *Japan World Econ.* **2**, 140–150 (2009).

[25] Kuroki, M. Suicide and unemployment in Japan: Evidence from municipal level suicide rates and age-specific suicide rates. *J. Socio-Econ.* **39**, 683–691 (2010).

[26] Browning, M. & Heinesen, E. Effect of job loss due to plant closure on mortality and hospitalization. *J. Health Econ.* **31**, 599–616 (2012). 22664774 10.1016/j.jhealeco.2012.03.001

[27] Classen, T. J. & Dunn, R. A. The effect of job loss and unemployment duration on suicide risk in the united states: A new look using mass-layoffs and unemployment duration. *Health Econ.* **21**, 338–350 (2012). 21322087 10.1002/hec.1719 PMC3423193

[28] Eliason, M. & Storrie, D. Does job loss shorten life?. *J. Hum. Resources* **44**, 277–302 (2009).

